# The quality of clinical practice guidelines for management of pediatric type 2 diabetes mellitus: a systematic review using the AGREE II instrument

**DOI:** 10.1186/s13643-018-0843-1

**Published:** 2018-11-15

**Authors:** Meha Bhatt, Ahmed Nahari, Pei-Wen Wang, Emily Kearsley, Nicole Falzone, Sondra Chen, Erin Fu, Yaanu Jeyakumar, Justyna Zukowski, Laura Banfield, Lehana Thabane, M. Constantine Samaan

**Affiliations:** 1 Department of Pediatrics, McMaster University, Hamilton, Ontario Canada; 2 Division of Pediatric Endocrinology, McMaster Children’s Hospital, Hamilton, Ontario Canada; 3 Department of Health Research Methods, Evidence, and Impact, McMaster University, Hamilton, Ontario Canada; 4 Department of Pediatrics, King Fahad Central Hospital, Jizan, Kingdom of Saudi Arabia; 5 Health Sciences Library, McMaster University, Hamilton, Ontario Canada; 6 Biostatistics Unit, St Joseph’s Healthcare, Hamilton, Ontario Canada; 7 Center for Evaluation of Medicines, St Joseph’s Healthcare, Hamilton, Ontario Canada

## Abstract

**Aims:**

Pediatric type 2 diabetes mellitus (T2DM) is a relatively new disease with increasing incidence corresponding to the obesity epidemic among youth. It is important for clinicians to have access to high-quality clinical practice guidelines (CPGs) for appropriate management of pediatric patients with T2DM. The objective of this systematic review was to evaluate overall quality of CPGs for the management of pediatric T2DM using the Appraisal of Guidelines for Research and Evaluation II (AGREE II) tool.

**Methods:**

We searched MEDLINE, Embase, CINAHL, Trip, National Guideline Clearinghouse, and grey literature to identify eligible CPGs. We also searched the webpages of national and international diabetes and pediatric organizations globally. We included CPGs from national and international diabetes and pediatric associations that were published as standalone guidelines for T2DM in children and adolescents (2–18 years of age). We also included pediatric and adult guidelines for type 1 diabetes if they included a section addressing T2DM management in children and adolescents. We retrieved the two most recent guidelines from each organization when available to assess change in quality over time. We excluded individual studies and systematic reviews that made treatment recommendations as well as CPGs that were developed for a single institution.

**Results:**

We included 21 unique CPGs in this systematic review. Of the included guidelines, 12 were developed or updated between 2012 and 2014. Five of all included CPGs were specific to pediatric populations. The analysis revealed that “Rigour of Development” (mean 45%, SD 28.68) and “Editorial Independence” (mean 45%, SD 35.19) were the lowest scoring domains on the AGREE II for the majority of guidelines, whereas “Clarity of Presentation” was the highest scoring domain (mean 72%, SD 18.89).

**Conclusions:**

Overall, two thirds of the pediatric T2DM guidelines were moderate to low quality and the remaining third ranked higher in quality. Low quality was especially due to the scores for the “Rigour of Development” domain, which directly measures guideline development methodology. It is important that future guidelines and updates of existing guidelines improve the methodology of development and quality of reporting in order to appropriately guide physicians managing children and adolescents with T2DM.

**Systematic review registration:**

PROSPERO CRD42016034187

**Electronic supplementary material:**

The online version of this article (10.1186/s13643-018-0843-1) contains supplementary material, which is available to authorized users.

## Introduction

Pediatric type 2 diabetes mellitus (T2DM) is a relatively new disease that is escalating due to the global rise in childhood obesity [1]. While close to 420 million adults have T2DM around the world, an increasing number of children are being diagnosed with T2DM and are transitioning to adult care, and this trend is likely to increase exponentially [2]. In the USA, there has been a 30.5% increase in the prevalence of pediatric T2DM between 2001 and 2009 [3].

On a mechanistic level, many adults present with risk factors for T2DM in the early years of life, and while genetics likely play a role in this diabetes risk, environmental and epigenetic factors lead the way in the development of diabetes in youth and young adults [4].

Pediatric T2DM often occurs among those who have existing medical conditions, e.g., transplant patients, and is associated with the development of comorbidities early in the course of the disease. These comorbidities include dyslipidemia, hypertension, non-alcoholic fatty liver disease, nephropathy, and obstructive sleep apnea and will likely contribute to adverse long-term cardiovascular outcomes in this population [3, 5]. While pediatric T2DM has been reported most frequently among certain ethnic groups (e.g., Indigenous North American communities, South Asians, and Latinos), a global spread of the condition has been reported [2]. This highlights the need for guidance on management developed for various populations. The increasing burden of T2DM has major implications for the quality of life, longevity, and healthcare system use in these patients globally [6]. Due to the relative novelty of this disease in pediatric populations and the importance of early management to avoid future complications, it is imperative that high-quality clinical practice guidelines (CPGs) are available for clinicians. CPGs are statements consisting of clinical recommendations that aid healthcare providers in decision-making and also inform policy-related and system-level decisions [7]. Systematically developed and evidence-based CPGs support best practices and have the potential to optimize patient care. Guidance for clinicians on managing pediatric T2DM has emerged in the past decade, although its quality has not been evaluated. Assessing the quality of CPGs for pediatric T2DM management will help determine areas requiring improvement in future guidelines and updates, in addition to providing information about overall quality to clinicians who consult these guidelines.

The objectives of this systematic review are to (1) evaluate the quality of published CPGs for the management of pediatric T2DM using the Appraisal of Guidelines for Research and Evaluation II (AGREE II) tool and (2) assess the change in quality of guidelines within an organization over time when applicable.

## Methods

This systematic review is registered with PROSPERO (registration number: CRD42016034187), and detailed methods are available in the published protocol [8]. This systematic review is reported according to the Preferred Reporting Items for Systematic Reviews and Meta-analyses (PRISMA) guidelines and a checklist is available as a supplementary material (Additional file 1) [9].

### Eligibility criteria

We included CPGs from national and international diabetes and pediatric associations that were published as standalone guidelines for T2DM in children and adolescents (2–18 years of age). We also included guidelines targeting T2DM in adults and pediatric type 1 diabetes if a section addressing T2DM management in children and adolescents was available. In order to determine the changes in quality of reporting over time, we retrieved the two most recent guidelines from each organization when available. For this review, we excluded all CPGs that were developed exclusively for use within a single institution and those that were not published or were under development at the time of our appraisal. We excluded individual systematic reviews and primary studies that provided recommendations on the management of T2DM. Guidelines addressing drug-induced and genetic forms of diabetes were also excluded [8].

### Search strategy

We searched MEDLINE, Embase, Cumulative Index to Nursing and Allied Health Literature (CINAHL), Turning research into practice (Trip) database, and the National Guideline Clearinghouse (guideline.gov) from inception through January 2016 to identify eligible CPGs. The database search was updated in August 2017. As we were aware of the Diabetes Canada guidelines launch in 2018, we updated the included guidelines accordingly. The complete search strategy used to retrieve guidelines is available as supplementary material (Additional file 2). Additionally, we extended our search to include guidelines that were not published in indexed journals. Four authors identified national and international diabetes and pediatric associations around the world and conducted a comprehensive search of their webpages for eligible guidelines. Earlier versions of CPGs were also obtained from the organization’s webpage, when available. We further extended our search by contacting the organizations in July 2017 to inquire about the two latest CPGs issued for pediatric T2DM, when it was unclear if they had earlier versions of guidelines from the webpage. The search was not limited by language, and we used Google Translate to appraise eligible guidelines that were not available in English. We judged the accuracy of the translation by back-translating the guidelines to the original language and assessed the publication for overall similarity. Our review required the use of Google Translate for guidelines in the following languages: Portuguese, Japanese, Italian, Greek, Latvian, Lithuanian, Dutch, Polish, Slovenian, and Thai. The overall accuracy of Google Translate has been examined by a 2011 study, finding acceptable scores (> 70 out of 100) for Portuguese, Italian, Greek, Dutch, and Slovenian [10]. However, the same study found lower scores (< 50 out of 100) for Japanese, Latvian, Lithuanian, and Polish and a poor score (0 out of 100) for Thai [10]. Another study on the use of Google Translate specifically during data extraction for systematic reviews examined agreement between reviewers, showing good agreement for Portuguese and fair agreement for Japanese, but did not examine other languages translated for our review [11]. Since we appraised the overall guideline based on broad criteria rather than extracting specific data points, we expected greater accuracy than when conducting data extraction for systematic reviews.

### Study selection and data extraction

Trained independent reviewers conducted title and abstract screening and full-text review of eligible studies in duplicate. Disagreements between reviewers were resolved by discussion to consensus, and an expert Pediatric Endocrinologist was consulted to resolve persisting disagreements. Two authors independently extracted general study characteristics (title, issuing society/association, country, year of publication) and details of management recommendations for T2DM including diagnostic criteria, treatment recommendations, screening tests for comorbidities, and frequency of screening.

### Quality appraisal

The AGREE II tool was used to evaluate the quality of CPGs included in this review [12]. The 23-item instrument evaluates guideline methodology and quality of reporting across six domains: (1) Scope and purpose, (2) Stakeholder involvement, (3) Rigour of development, (4) Clarity of presentation, (5) Applicability, and (6) Editorial independence. Each domain consists of a number of statements pertaining to the topic, and individual appraisers rank each statement on a 7-point Likert scale, varying from strongly disagree to strongly agree (1 to 7, respectively). Domain scores from individual appraisers are summed and then scaled to a percentage of the maximum possible score for that domain. The maximum and minimum possible scores are determined based on the number of statements pertaining to each domain and the number of appraisers, and the following formula is used to calculate the scaled domain score: $\frac{\mathrm{Obtained}\ \mathrm{score}-\mathrm{Minimum}\ \mathrm{possible}\ \mathrm{score}}{\mathrm{Maximum}\ \mathrm{possible}\ \mathrm{score}-\mathrm{Minimum}\ \mathrm{possible}\ \mathrm{score}}$. Therefore, higher scores indicate higher quality. The domain scores are independent and are not aggregated into a single quality score. Additionally, the instrument requires appraisers to provide an overall judgment of guideline quality and whether the guideline can be recommended for use with possible responses of “yes,” “yes with modifications,” and “no.”

The AGREE II developers recommend at least two but preferably four appraisers in order to increase the reliability of the assessment [13]. In the current systematic review, four trained independent appraisers evaluated eligible CPGs using the AGREE II tool. When guidelines included a pediatric T2DM section but also addressed adult diabetes or pediatric type 1 diabetes, the overall guideline methods were used to assess stakeholder involvement, rigour of development, and editorial independence whereas the remaining domains were assessed specifically for the pediatric T2DM chapter.

### Data synthesis

All statistical analyses were conducted using SPSS Statistics Version 23 (IBM SPSS Statistics for Macintosh, Version 23.0, Armonk, NY). We calculated agreement between reviewers for guideline eligibility using Cohen’s kappa statistic and inter-rater reliability using the intraclass correlation coefficient (ICC). We summarized general characteristics of CPGs using descriptive statistics.

For each included CPG, we calculated a quality score for each of the six AGREE II domains by summing up the appraisers’ scores for individual items and scaling the total as a percentage of the maximum possible score for that domain [12]. The overall scores of the included CPGs are presented for each AGREE II domain using summary statistics (mean, SD). The AGREE II domains were evaluated independently, such that the appraisal scores for the domains were not aggregated. We conducted paired *t*-tests to compare mean domain scores for the earlier and latest versions of guidelines, when available, to determine change in quality over time. The level of significance was set at alpha = 0.05.

## Results

**Fig. 1:**
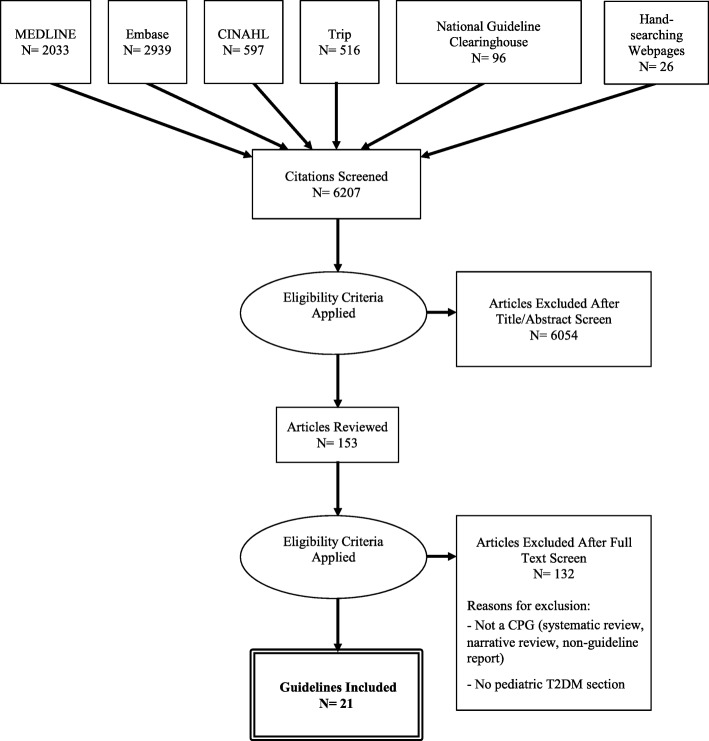
Flow diagram for selection process of pediatric type 2 diabetes guidelines

Following screening of 6207 citations, we reviewed 153 articles and included 21 unique CPGs published by national and international organizations [14–34]. Reasons for exclusion during full text review were non-CPG articles (i.e., systematic reviews, narrative reviews, non-guideline reports) and lack of a pediatric T2DM section within guideline documents. Figure 1 shows the flow diagram for study inclusion. We also identified earlier versions for 7 of 21 included guidelines [35–41]. The kappa for full-text review was 0.87 (95% confidence interval 0.77 to 0.96), indicating excellent inter-rater agreement [42].

### Characteristics of included guidelines

Table 1 outlines the basic characteristics of the included guidelines, and Table 2 summarizes the screening and management recommendations from each guideline. The abstraction of diagnostic criteria of T2DM assumed that HbA1c is not used alone for the diagnosis of pediatric patients.

**Table 1:** Basic characteristics of included guidelines

	Number	Percent
Year of publication		
2006–2008	1	4.8
2009–2011	1	4.8
2012–2014	9	42.9
2015–2018	10	47.6
Continent of published guidelines		
Europe	10	47.6
Asia	4	19.0
North America	3	14.3
Africa	1	4.8
South America	1	4.8
International	2	9.5
Organization publishing guideline		
Medical society	17	81.0
Government	4	19.0
Guideline specific to pediatric population?		
Yes	5	23.8
No	16	76.2

**Table 2:** Screening, diagnostic criteria and management recommendations for pediatric type 2 diabetes

Guideline characteristics			Treatment recommendations				
Organization	Year	Country	Criteria for screening	Frequency of screening	Recommended screening tests	Diagnostic criteria	Recommendation of healthcare delivery
Brazilian Diabetes Society	2015/2016	Brazil	The diagnosis of T2DM in childhood should be made taking into account clinical criteria such as age and sex of the patient, obesity and positive family history for T2DM. Following these criteria, doubtful cases, especially those with initial ketoacidosis should be evaluated further. Due to high miscegenation in Brazil, there are no data to consider race as a risk factor. However, this guideline also lists the ADA recommendations: Screening for DM2 in childhood, should include every obese child with BMI greater than the 85th percentile for age and gender, or weight greater than 120% of the ideal weight for height, presenting with two or more of the following risk factors: (1) positive family history for DM2 in first or second degree relatives; (2) Certain ethnic backgrounds (American Indians, African Americans, Hispanics, Asian/ Pacific Islander); (3) IR signals or conditions associated with IR (AN, hypertension, dyslipidemia, Polycystic ovary syndrome.	Every 2 years, beginning after the age of 10	-RPG, FPG, OGTT -Evaluation of beta cell function by C-peptide -The detection of markers of pancreatic islet autoimmunity using autoantibodies including anti-GAD, Anti-IA2, ICA and IAA.	Consider the diagnosis of T2DM in 10–18 year old patients with the following criteria: • Overweight or obesity (respectively for gender and age with BMI percentile ≥ 85th percentile) • Strong family history of T2DM • Substantial residual capacity of insulin secretion Diagnosis (proven by normal or high concentration of insulin and C-peptide) • Insidious onset of disease • IR (clinical evidence of PCOS and NAFLD) • Exclusion of autoimmune diabetes (autoantibodies typically associated with type 1 diabetes are not detected). • the diagnosis is defined biochemically as: -Fasting glycemia (defined as absence of caloric intake during a minimum period of 8 h) ≥ 126 mg/ dL (≥ 7.0 mmol/L) • Plasma glucose on OGTT at 2 h post challenge of ≥200 mg / dL (≥ 11.1 mmol/L), and the test is carried out in accordance with WHO recommendation, using Glucose solution containing 75 g of anhydrous glucose Diluted in water • Random plasma glucose ≥ 200 mg /dL (≥ 11.1 mmol/L), with typical symptoms of hyperglycemia (polyuria, polydipsia, loss of weight).	*Physical activity*: Children and adolescents with T2DM Should be encouraged to perform moderate to intense physical activity for at least 60 min daily. *Diet*: In the diet recommendation for children with T2DM, or at the time of or during treatment, using recommendations of the Academy of Nutrition Pediatric Weight Management Practice Guidelines *Screen time*: Non-academic (e.g., television, video games) is limited to 2 h/day *Addressing psychosocial needs*: Not reported *Medical and surgical therapies*: Metformin should be the drug of first choice for young people and adolescents at the time of diagnosis and beyond, but to be combined with lifestyle modification, including nutritional guidance and physical activity. Introduction of insulin treatment should be done in children and adolescents who have ketosis or diabetic ketoacidosis, in patients in whom distinction between type 1 diabetes versus T2DM is not clear, and where T2DM patients present with random plasma glucose or venous value ≥ 250 mg/dL (> 13.9 mmol/L) or HbA1c > 9%. *Target HbA1c*: < 7%; if not obtained, it is recommended to intensify the treatment.
Diabetes Canada	2018	Canada	Risk factors for the development of type 2 diabetes in children include a history of type 2 diabetes in a first- or second-degree relative, being a member of a high-risk population (e.g., people of African, Arab, Asian, Hispanic, Indigenous or South Asian descent), obesity, impaired glucose tolerance, polycystic ovary syndrome (PCOS), exposure to diabetes in utero, acanthosis nigricans, hypertension, dyslipidemia, and NAFLD.	Every 2 years (In Indigenous children, this should begin at 10 years of age or with puberty, whichever sooner)	Use a combination of an A1C and a FPG or random plasma glucose in children and adolescents with any of the following conditions (three or more risk factors in non-pubertal or two or more risk factors in pubertal children): • Obesity (BMI ≥ 95th percentile for age and gender) • Member of a high-risk ethnic group (e.g., African, Arab, Asian, Hispanic, Indigenous or South Asian descent) • Family history of T2DM and/or exposure to hyperglycemia in utero • Signs or symptoms of IR(including acanthosis nigricans, hypertension, dyslipidemia, PCOS and NAFLD [ALT >3× upper limit of normal or fatty liver on ultrasound)) • Impaired fasting glucose or impaired glucose tolerance • Use of atypical antipsychotic medications If there is a discrepancy between the A1C and FPG or random plasma glucose, testing may be repeated or a 2-h OGTT (1.75 g/kg; maximum 75 g) may be performed -OGTT (1.75 g/kg anhydrous glucose; maximum 75 g) may be used as a screening test in very obese children (BMI ≥99th percentile for age and gender) or those with multiple risk factors	-Fasting glycemia defined as FPG of ≥ 126 mg/dL (≥ 7.0 mmol/L) -Plasma glucose on OGTT at 2 h post challenge of ≥ 200 mg/dL (≥ 11.1 mmol/L), -Random plasma glucose ≥ 200 mg/ dL (≥ 11.1 mmol/L) with typical symptoms of hyperglycemia (polyuria, polydipsia, Loss of weight).	*Physical activity*: Recommend (in the absence of direct evidence for this population) that children strive to achieve the same activity level recommended for children in general: 60 min daily of moderate-to vigorous physical activity *Diet*: Not reported *Screen time*: Limiting screen time to 2 h/day *Psychosocial issues*: Psychological issues, such as depression, binge eating and smoking cessation, need to be addressed and interventions offered. *Medical and surgical therapies*: Insulin is required in those with severe metabolic decompensation at diagnosis (e.g., DKA, HbA1c of ≥ 9.0%, symptoms of hyperglycemia) but may be successfully weaned once glycemic targets are achieved, particularly if lifestyle changes are effectively adopted. Metformin has been shown to be safe in adolescents for up to 16 weeks, reducing HbA1c by 1.0–2.0% and lowering FPG. Side effects are similar to those seen in adults. Glimepiride has also been shown to be safe and effective in adolescents for up to 24 weeks. The experience of bariatric surgery in adolescents with type 2 diabetes is very limited with specific eligibility criteria (BMI > 35 kg/m^2^, Tanner stage IV-V, and skeletal maturity). *Target HbA1c*: ≤ 7%
German Diabetes Association (Deutsche Diabetes Gesellschaft)	2015	Germany	All overweight children (BMI > 90th percentile) age 10 years or older who have two or more risk factors: • A close blood relation has type 2 diabetes • Membership of a group with elevated risk (e. g. East Asians, Afro-Americans, Hispanics) • Extreme obesity (BMI > 99.5th Percentile) • Signs of IR or of changes associated with it (arterial hypertension, dyslipidemia, elevated transaminases, PCOS, AN).	Not reported	FPG, RPG, OGTT	-Fasting glucose: ≥126 mg/dL (≥ 7.0 mmol/L), -OGTT: 2-h level ≥ 200 mg/dL (≥11.1 mmol/L). if patient asymptomatic, repeat the test -Indications for distinguishing between type 2 and type 1 diabetes can improve by the following additional laboratory tests: • C-peptide • Autoantibodies (GAD, IA2, ICA, IAA)	*Physical activity:* Not reported *Diet:* Not reported *Screen time:* Not reported *Psychosocial issues:* Particularly in adolescents, to look for signs of eating disorders and affective disorders (anxiety, depression). If there is a psychiatric disorder, a psychiatrist or psychologist should be consulted *Medical and surgical therapies*: If initial HbA1c is ≥9% with blood glucose ≥250 mg/dL (≥13.9 mmol/L) and if signs of absolute insulin deficiency, initial insulin therapy is recommended. -Oral hypoglycemic agents have to be used when glycemic control by lifestyle intervention proves to be insufficient. Metformin ± insulin is needed. *Target HbA1c:* < 7%. In addition, treatment aim is to achieve a fasting glucose level under 126 mg/dL (< 7.0 mmol/L)
Hellenic Diabetes Federation	2013	Greece	Not reported for children. Some criteria that may apply to children: • Waist circumference ≥ 102 cm (male) and ≥ 88 cm (female) • Body mass index ≥ 30 kg/m^2^ • Family history of diabetes in parents, siblings, children • History of hypertension • History of gestational diabetes • Polycystic ovarian syndrome	Not reported for children. For adults, if normal FPG, recheck every 3 years	FPG, RPG, OGTT	FPG of ≥126 mg/dL (≥ 7.0 mmol/L) -Plasma glucose on OGTT at 2 h post challenge of ≥ 200 mg/dL (≥ 11.1 mmol/L), -Random plasma glucose ≥ 200 mg/dL (≥ 11.1 mmol/L) with symptoms	*Physical activity*: 30 min of exercise 5 times a week *Diet*: Managing carbohydrate intake *Screen time*: Not reported *Psychosocial issues*: Not reported *Medical and surgical therapies*: **•** Immediate initiation of insulin if there is significant hyperglycemia, metabolic deregulation, and hyperketonemia. • Metformin • Sulfonylureas in teens may be tried if metformin is not effective *Target HbA1c*: < 7.5% in children, ≤ 7.0% in teens
Associazione Medici Diabetologi/Societa Italiana di Diabetologia	2016	Italy	-At-risk children with BMI between the 85th–95th percentile: -Positive family history of T2DM -Early cardiovascular disease in family -AN -Hypertension -Dyslipidemia -PCOS -Children with BMI ≥ 95th percentile Distinction between type 1 versus type 2 diabetes can be difficult and may need to be based on the presence of autoantibodies, C-peptide and insulin levels	Not reported	- FPG, RPG, OGTT -HbA1c recommended, however the screening section is not reporting on the non-use of HbA1c in pediatric populations	-Fasting glycemia defined as FPG of ≥ 126 mg/dL (≥ 7.0 mmol/L) -Plasma glucose on OGTT at 2 h post challenge of ≥ 200 mg/ dL (≥ 11.1 mmol/L), -Random plasma glucose ≥ 200 mg/ dL (≥ 11.1 mmol/L) with typical symptoms of hyperglycemia. If asymptomatic, to repeat testing on a separate day	Using ADA recommendations for lifestyle management *Medical and surgical therapies*: Metformin is the drug of choice for diabetes type 2 without ketosis in diabetic adolescents. Insulin therapy should be initiated in case of marked hyperglycemia with ketosis. There is no indication for the use of sulfonylureas in pediatric patients. *Target HbA1c*: Not specifically reported, assumed to follow ADA recommendations
Japan Diabetes Society	2013	Japan	Not reported	Not reported	-FPG, RPG, OGTT -HbA1c recommended, however the screening section is not reporting on the non-use of HbA1c in pediatric populations	Fasting glycemia defined as FPG of ≥ 126 mg/dL (≥ 7.0 mmol/L) -Plasma glucose on OGTT at 2 h post challenge of ≥ 200 mg/ dL (≥ 11.1 mmol/L), -Random plasma glucose ≥ 200 mg/ dL (≥ 11.1 mmol/L) with typical symptoms of hyperglycemia	*Physical activity*: Every effort should be made to increase physical activity levels and energy expenditure in pediatric/adolescent patients to improve IR. *Diet*: Recommendations are aimed at providing patients with necessary and sufficient energy for normal development and growth, adjusted by age and sex. In obese patients, the amount of energy intake should be adjusted to 90–95% of the energy required for ideal body weight. *Screen time*: Not reported *Psychosocial issues*: Not reported *Medical and surgical therapies*: In patients who are not insulin-dependent and whose glycemic goals cannot be achieved with diet/exercise therapy alone within 2–3 months, oral glucose-lowering agents should be initiated. In patients with ketoacidosis or those in whose adequate glycemic control cannot be achieved with oral hypoglycemic agents, insulin therapy should be used. Patients should also be treated for hypertension, dyslipidemia and other comorbidities *Target HbA1c*: < 7%
Latvijas Diabēta Asociācija/Latvijas Endokrinologu Asociācija	2007	Latvia	BMI with regards to age and gender is > 85% and two of the following risk factors: -Family history of first or second degree relatives with T2DM -Populations with increased risk (e.g., African-Americans, American Indians, Asian and Pacific Islander population) -Any of the following symptoms of IR: AN, dyslipidemia, or PCOS	10 years or at the beginning of puberty whichever sooner, then every 2 years	FPG, RPG, OGTT.	Diagnosis of T2DM if random glucose ≥11.1 mmol/l or glucose level 2 h post OGTT ≥ 11.1 mmol/l. If fasting blood glucose on two repeat tests ≥7.0 mmol/l	*Physical activity*: At least 15–30 min a day. *Diet*: Total daily caloric based the age and body weight *Screen time*: Not reported *Psychosocial issues*: Not reported *Medical and surgical therapies*: • Metformin is the first drug of choice (maximum dose of 2000 mg/day) • Insulin therapy, using appropriate insulin regimen. • If the fasting glucose > 270 mg/dL (> 15 mmol/L), ketosis or ketoacidosis, and HbA1c > 9%, then use insulin ± metformin. • If there is diabetic ketosis, but HbA1c 7–9%, then treatment with metformin and suggest to follow diet recommendations as well as regular physical activity. Recommend clinic visits every 3 months to determine the HbA1c. If HbA1c is > 7% after 6 months of treatment, to begin insulin therapy. • If HbA1c is < 7%, then suggest to follow diet and physical activity recommendations. If, after 3 months of non-pharmacological treatment HbA1c > 7%, use metformin. *Target HbA1c*: ≤ 7.5% for 6–12 years of age ≤ 6.5% in children over 12 years of age
Diabetasgaires	2014	Lithuania	Reference criteria from International Diabetes Federation	Not reported	-Pancreatic autoantibodies should be tested for autoimmunity in suspected cases of T2DM to clarify the diagnosis -Pancreatic antibodies should be tested for all obese / overweight children > 13 years, when suspicion of clinical type 1 diabetes (weight loss, ketosis / ketoacidosis) -With T2DM diagnosis, it is recommended to measure blood pressure, fasting lipids, test urine for microalbuminuria and do dilated eye exam to examine the retina. Monitoring and treatment guidelines for hypertension, dyslipidemia, microalbuminuria and retinopathy are the same as type 1 diabetes. Specific problems such as PCOS, and various diseases associated with obesity, such as sleep apnea, NAFLD, Orthopedic complications and psychosocial problems must be also tested for.	Diabetes mellitus is diagnosed by detecting at least one of the criteria: FPG ≥ 7.0 mmol/L and/or blood glucose 2 h post 75 g of glucose ≥ 200 mg/dL (≥ 11.1 mmol/L). Test for pancreatic antibodies recommended	*Physical activity*: Recommended lifestyle changes, no further details *Diet*: Recommended lifestyle including nutritional changes, no further details *Screen time*: Not reported *Psychosocial issues*: Not reported *Medical and surgical therapies*: Metabolically stable patients first-line drug is metformin (approved the use for those 10 years and older). If within 3 months failed to reduce or maintain HbA1c < 7% to treat with insulin. In the beginning, in the presence of metabolic instability, it may be necessary to start treatment with insulin *Target HbA1c*: < 7%
Malaysian Ministry of Health	2015	Malaysia	-Symptomatic individuals should be screened for diabetes Asymptomatic individuals should be screened if they are -Overweight (BMI > 85th percentile for age and sex, or weight > 120% of ideal body weight) and Have two or more of the following risk factors: -Family history of T2DM in first- or second-degree relatives. -Signs and conditions associated with IR (AN, hypertension, dyslipidemia, PCOS). -Maternal history of gestational diabetes	Initiated at 10 years of age or at onset of puberty, whichever sooner	Fasting insulin and C-peptide has been used to aid in the diagnosis. However, their measurement should be interpreted with caution due to considerable overlap between type 1 diabetes, T2DM and monogenic diabetes at onset and within 2 years of diagnosis.	FPG, OGTT, Random blood glucose In symptomatic individual, one abnormal glucose value is diagnostic. In asymptomatic individual, two abnormal glucose values are required. Diagnostic values: venous blood samples demonstrating • FPG ≥ 7.0 mmol/L • Random or 2 h post OGTT plasma glucose ≥ 11.1 mmol/L.	*Physical activity*: 150 min per week and/or at least 90 min/week of vigorous aerobic plus at least two sessions per week of resistance exercise *Diet*: Nutrition recommendations following the adult guidelines: -Total carbohydrate intake should be measured to help the patient understand content **-**Fat content should be 25–35% of daily intake -Protein intake 15–20% of daily intake *Screen time*: Not reported *Psychosocial issues*: Regular screening for eating disorders and depression *Medical and surgical therapies*: Among all the drugs currently used to treat T2DM in adults, only metformin and insulin are FDA approved for use in adolescents. Metformin should be started with 500 mg daily for 7 days. Gradual dose increment by 500 mg once a week over 3–4 weeks until the maximal dose of 1000 mg twice daily. Insulin may be required for initial metabolic control. Transition from insulin to metformin can usually be made when metabolic stability is reached. This may take 2–6 weeks. Long-acting or intermediate acting insulin may be added at a dose of 0.5 units/kg/day, but can be increased to achieve glycemic targets. *Target HbA1c*: < 6.5%
Netherlands Diabetes Federation	2015	Netherlands	In the Netherlands, no systematic screening is done.	Screening criteria are not applicable since systematic screening is not recommended.	Screening tests are not applicable since systematic screening is not recommended.	Fasting glucose: ≥ 126 mg/dL (≥ 7.0 mmol/L), -OGTT: 2-h level ≥ 200 mg/dL (≥ 11.1 mmol/L). if patient asymptomatic, repeat the test	*Physical activity*: Recommend 60 min/day of moderately vigorous physical activity. Intensive lifestyle programs with professional guidance in the areas of nutrition therapy, exercise and behavioral change in the treatment of overweight/obesity is preferred. *Diet*: Advise is blended for type 1 and type 2 diabetes. Counsel patients to reduce the intake of refined carbohydrates and sweetened drinks. Suggest individualized carbohydrate intake, and to avoid elimination of food items to curb the risk of eating disorders. Fat intake should be defined as per healthy age-based intake recommendations. The use of plant sterols in children over 5 years is acceptable if LDL is elevated. For children with diabetes, there is not enough evidence to recommend an ideal amount of protein for optimizing glucose and improving cardiovascular risk. Micronutrients The recommendation for vitamins, minerals and trace elements for children with diabetes is the same as for healthy children. Vitamin D supplementation is necessary in young children It is recommended that wherever possible, to replace processed products by unprocessed products. *Screen time*: Not reported *Psychosocial issues*: Not reported specifically, but encourages the focus on a positive body image and to avoid food restrictions to prevent eating disorders. Nutritional advice should be discussed with the whole family and tailored to the child’s cultural and individual needs. *Medical and surgical therapies*: lifestyle intervention with insulin therapy *Target HbA1c*: ≤ 7%
Polskiego Towarzystwa Diabetologicznego	2015	Poland	Obese children above 10 years of age or puberty, whichever is earlier (BMI > 95%)	Every 2 years in children > 10 years	Random blood glucose, FPG, or OGTT	• FPG ≥ 7.0 mmol/l; or • Venous plasma glucose ≥ 11.1 mmol/L (≥ 200 mg/dl) at 2 h after a 75 g OGTT or random measurement if symptomatic -If asymptomatic, or symptomatic but blood glucose < 200 mg/dL (< 11.1 mmol/L) needs two tests to establish the diagnosis at different times	*Physical activity*: Moderate physical activity > 60 min/day *Diet*: Maintain proper balance of calories and gradual reduction of carbohydrates, up to 45–50% daily caloric intake; restrict sugars up to 10% daily caloric requirement *Screen time*: Not reported *Psychosocial issues*: Provide psychological care with children with diabetes and their families with support; screening for depression and eating disorders should be performed in all patients at 1–2 years intervals *Medical and surgical therapies*: Treatment of choice is metformin and/or insulin; if HbA1c < 9, and no symptoms of disease or acidosis, metformin; should be used. If symptomatic, give metformin and basal insulin. If ketotic, start insulin similar to type 1 diabetes (short and intermediate or long acting insulin) *Target HbA1c*: ≤6.5%
Scottish Intercollegiate Guidelines Network^*^	Updated 2017	Scotland	Not reported	Not reported	FPG, OGTT	The clinical diagnosis of diabetes is often indicated by the presence of symptoms such as polyuria, polydipsia, and unexplained weight loss, and is confirmed by measurement of hyperglycemia. • FPG ≥ 7.0 mmol/l; or • Venous plasma glucose ≥ 11.1 mmol/L (≥ 200 mg/dl) at 2 h after a 75 g OGTT	*Physical activity*: People with type 2 diabetes should be encouraged to participate in physical activity or structured exercise to improve glycemic control and cardiovascular risk factors. Exercise and physical activity (involving aerobic and/or resistance exercise) should be performed on a regular basis. *Diet*: Structured education program are recommended. *Screen time*: Not reported *Psychosocial issues*: Children should be offered psychological interventions (motivational interviewing, goal setting skills and Cognitive Behavioral Therapy) to improve glycemic control *Medical and surgical therapies*: Metformin and insulin therapy are the mainstay of treatment. *Target HbA1c*: 7% is reasonable to reduce risk of microvascular disease and macrovascular disease; a target of 6.5% may be appropriate at diagnosis
Singapore Ministry of Health	2014	Singapore	Children and adolescents with suspected diabetes; screening for T2DM in asymptomatic children and adolescents is not recommended	Not reported	FPG, Casual plasma glucose, OGTT	Casual plasma glucose of ≥ 200 mg/dL (≥ 11.1 mmol/L); FPG of ≥126 mg/dL (≥ 7 mmol/L); 2 h post challenge plasma glucose ≥200 mg/dL (≥ 11.1 mmol/L)	*Physical activity*: Children may initially be treated with lifestyle modification (diet and exercise) unless they are symptomatic or severely hyperglycemic; lifestyle changes in diet and exercise should be recommended for all children with type 2 diabetes mellitus and continued, even after addition of pharmacologic therapy *Diet*: Not reported specifically for children *Screen time*: Not reported *Psychosocial issues*: Psychoeducational interventions recommended *Medical and surgical therapies*: Metformin may be started as the first-line oral agent if blood glucose targets are not achieved with lifestyle intervention. Insulin should be started if oral agents fail to attain target control. If monotherapy with metformin over 3–6 months has failed, insulin should be added to the treatment *Target HbA1c*: < 7.5%
Endodiab	2016	Slovenia	Obese adolescents BMI > 98th percentile; overweight adolescents BMI > 91st percentile with two additional criteria: -Presence of T2DM in a relative; −Exposure to gestational diabetes -Belonging to racial/ethnic groups more likely to have T2DM -Clinical signs of IR	Every 2–3 years	Random blood glucose, fasting insulin and OGTT	Random blood glucose ≥ 200 mg/dL(> 11.1 mmol/L); Symptoms of diabetes (polyuria, polydipsia, unexplained weight loss) and plasma glucose ≥ 200 mg/dL (> 11.1 mmol/L); FPG ≥ 7 mmol/L glucose levels 2 h post OGTT > 11.1 mmol/l	*Physical activity*: Begin with the implementation of the program for lifestyle changes including addressing the diet and psychologist *Diet*: Not reported specifically for children *Screen time*: Not reported *Psychosocial issues*: Begin with the implementation of the program for lifestyle changes including addressing the diet and psychologist *Medical and surgical therapies*: If HbA1c is < 9%, start with lifestyle intervention and add Metformin. Start with a dose of 500 mg/day, increase by 500 mg/week to a maximum dose of 1000 mg two times a day. If within 3–4 months metformin does not lead to HbA1c < 6.5%, add basal insulin. -If patients are metabolically unstable or HbA1c > 9%, insulin treatment is needed. If not acidotic, insulin and metformin can start simultaneously. After 2–6 weeks can switch to monotherapy with metformin. -In overweight adolescents with BMI > 40 kg/m^2^ who are pubertal and > 15 years old, with failed conservative approach of dietary therapy and physical activity and metformin for more than 6 months, possible treatment of bariatric surgery is considered. *Target HbA1c*: < 6.5%
Society for Endocrinology, Metabolism and Diabetes of South Africa	2017	South Africa	Little evidence justifies systematic screening of asymptomatic children for T2DM. However, opportunistic testing should be considered in overweight patients (BMI ≥ 85%) who satisfy at least 2 of the following criteria: -Family history of T2DM in first or second degree relative -High risk ethnicity -Signs or conditions associated with IR -If BMI ≥ 99th percentile	Initial screening may begin at 10 years or at onset of puberty, whichever occurs earlier, and should be performed every 2 years	FPG is preferred, but if borderline do OGTT. Symptoms of diabetes and a fasting or random plasma glucose	OGTT 2 h post-challenge plasma glucose ≥ 200 mg/dL (≥ 11.1 mmol/L); or FPG ≥ 126 mg/dL (≥ 7.0 mmol/L); or random plasma glucose ≥ 200 mg/dL (≥ 11.1 mmol/L); HbA1c ≥ 6.5%	*Physical activity*: The aim is to increased exercise capacity *Diet*: Not reported *Screen time*: Not reported *Psychosocial issues*: Not reported *Medical and surgical therapies*: Treat with insulin if patient presents with ketosis, acidosis and dehydration. Metformin can be added later with a 500 mg daily dose at first, and then increase to a maximum dose of 1000 mg twice daily over 3–4 weeks. In an otherwise well, less symptomatic child, metformin is the treatment of choice *Target HbA1c*: < 7%
Diabetes Association of Thailand	2014	Thailand	Children and adolescents aged 10 years and over who are obese and have 2 risk factors out of the following: -Have parents or siblings with diabetes -Have high blood pressure (BP ≥ 130/85 mmHg) -Acanthosis nigricans	Start screening at ≥ 10 years	FPG, random plasma glucose, OGTT	Symptomatic or asymptomatic blood glucose levels ≥ 200 mg/dL (≥ 11.1 mmol/L); OGTT 2-h plasma glucose ≥ 200 mg/dL (≥ 11.1 mmol/L); FPG ≥ 126 mg/dL (≥ 7.0 mmol/L)	*Physical activity*: General recommendation for exercise. *Diet*: Determine food and energy requirements by age; food consists 50–60% carbohydrates, 25–30% fat and 15–20% protein; sweetened drinks should be less than 5% and should be consumed with high fiber foods; suggest eating more vegetable fats than animal fat. *Screen time*: Not reported *Psychosocial issues*: Build understanding and self-care experience; practice and help with psychological adjustment for patients and parents; provide support, adaptation, and family preparedness *Medical and surgical therapies*: Aim for weight loss of 5–10%. Insulin therapy if HbA1c is > 9% or blood glucose ≥11.1 mmol/L. with improvement, start metformin 250 mg daily for 3–4 days, then increased within 3–4 weeks, max amount 1000 mg twice per day. Reduce insulin by 10–20% each time metformin was adjusted up to target and then discontinue insulin, most take 2–6 weeks to stop the insulin injection. *Target HbA1c*: < 7.5%
National Institute for Health and Care Excellence	Updated 2016	United Kingdom	Consider T2DM in children and young people with the following risk factors: -Strong family history of type 2 diabetes -Obese at presentation -Black or Asian family origin -Have no insulin requirement, or have an insulin requirement of less than 0.5 units/kg/day after the partial remission phase -Show evidence of insulin resistance (for example, acanthosis nigricans)	Not reported	Random plasma glucose test, FPG, OGTT OGTT is not usually necessary or appropriate for children and young people who present with symptoms (thirst, polydipsia (increased drinking), polyuria (increased urine output), recurrent infections and weight loss, hyperglycemia, marked glycosuria and ketonuria).	Random plasma glucose concentration ≥ 200 mg/dL (≥ 11.1 mmol/L); or FPG ≥ 126 mg/dL (≥ 7.0 mmol/L); or random plasma glucose concentration ≥ 200 mg/dL (≥ 11.1 mmol/L) post OGTT	*Physical activity*: Advise children or young persons who are overweight or obese about the benefits of physical activity and weight loss, and provide support towards achieving this *Diet*: Explain to child or young person and their family members or carers how healthy eating can help to reduce hyperglycemia and cardiovascular risk, and promote weight loss; provide dietary advice in a sensitive manner, taking into account the difficulties that many people encounter with weight reduction, and emphasize the advantages of healthy eating for blood glucose control and avoiding complications. To take into account social and cultural considerations when providing advice on dietary management. Encourage people with type 2 diabetes to eat at least 5 portions of fruit and vegetables each day *Screen time*: Not reported *Psychosocial issues*: Diabetes teams should be aware that children and young people with T2DM have a greater risk of emotional and behavioral difficulties; offer emotional support after diagnosis; offer timely and ongoing access to mental health professionals with an understanding of diabetes because they may experience psychological problems or psychosocial difficulties that can impact the management of diabetes and wellbeing; offer screening for anxiety and depression to children and young people who have persistently suboptimal glycemic control *Medical and surgical therapies*: Offer metformin from diagnosis; offer bariatric surgery in centers that have dedicated pediatric facilities for children and young people with diabetes; all centers should have written protocols on safe surgery for children and young people. *Target HbA1c*: ≤6.5%
American Diabetes Association	2016	United States	Children and adolescents who are overweight or obese and who have two or more of the following risk factors for diabetes: -Family history of type 2 diabetes in first- or second-degree relative -Race/ethnicity (Native American, African American, Latino, Asian American, Pacific Islander) -Conditions associated with insulin resistance (acanthosis nigricans, hypertension, dyslipidemia, PCOS, or small-for-gestational age) -Maternal history of diabetes	3 year intervals beginning at 10 years or onset of puberty, whichever is earlier	Random plasma glucose, FPG and OGTT	Fasting plasma glucose ≥126 mg/dL (≥ 7.0 mmol/L); or 2 h post OGTT plasma glucose ≥200 mg/dL (≥ 11.1 mmol/L); or random plasma glucose ≥200 mg/dL (≥ 11.1 mmol/L)	*Physical activity*: To exercise regularly and maintain a healthy weight *Diet*: Eat a balanced diet; nutrition recommendations should be culturally appropriate and sensitive to family resources *Screen time*: Not reported *Psychosocial issues*: Not reported *Medical and surgical therapies*: Presentation with ketosis or ketoacidosis requires a period of insulin therapy until fasting and postprandial glycemia have been restored to normal or near-normal; insulin is used if HbA1c is > 9% or if blood glucose levels are > 250 mg/dL (13.9 mmol/L). Metformin therapy may be used after resolution of ketosis/ketoacidosis or in stable patients at diagnosis. *Target HbA1c*: < 7.5%
American Academy of Pediatrics	2013	United States	Overweight or obesity; strong family history of T2DM; the presence of IR	Not reported	HbA1c test (not alone), fasting plasma glucose test, oral glucose tolerance test, random plasma glucose test	HbA1c ≥6.5%; fasting plasma glucose ≥ 126 mg/dL; or 2 h plasma glucose OGTT ≥ 200 mg/dL (≥ 11.1 mmol/L); or random plasma glucose ≥ 200 mg/dL (≥11.1 mmol/L) with symptoms	*Physical activity*: Clinicians should initiate a lifestyle modification program, including nutrition and physical activity. Recommend engagement in moderate-to-vigorous exercise for at least 60 min daily *Diet*: Not reported *Screen time*: Limit non-academic screen time to less than 2 h a day *Psychosocial issues*: Not reported *Medical and surgical therapies*: Insulin is used if HbA1c is > 9% or if blood glucose levels are > 250 mg/dL (13.9 mmol/L). Start metformin as first-line therapy for children and adolescents at the time of diagnosis with lifestyle intervention. *Target HbA1c*: < 7.0%
International Society for Pediatric and Adolescent Diabetes (ISPAD)	2014	International	Obese at-risk youth	Not reported	Tests for fasting plasma glucose, OGTT, or casual plasma glucose	Diagnosis can be made based on fasting glucose, 2-h post challenge glucose, or HbA1c; in the absence of symptoms, testing should be confirmed on a different day; fasting plasma glucose of ≥ 126 mg/dL (≥ 7 mmol/L), or 2 h-post challenge plasma glucose of ≥ 200 mg/dL (11.1 mmol/L); or casual plasma glucose ≥ 200 mg/dL (≥ 11.1 mmol/L); or HbA1c > 6.5% (not alone, use in diagnosis of T2DM is controversial)	*Physical activity*: Lifestyle change should be initiated at the time of diagnosis of T2DM; increased physical activity to at least 60 min per day of moderate-to-vigorous activities. Activity prescriptions should be developed and should be sensitive to family environment and resources. *Diet*: Reduced carbohydrate and fat intake, increased fruit and vegetable intake, elimination of sugar-sweetened beverages and juice, reduce intake of processed and prepackaged foods, reduce intake of foods with refined sugars, reduce eating out. Change recommended in eating behaviors. *Screen time*: Reduction in sedentary time including TV, computer related activities, texting, and video games, screen time limited to < 2 h/day *Psychosocial issues*: Should be assessed for depression; identified patients should be referred to appropriate mental healthcare providers experienced in addressing depression in youth *Medical and surgical therapies*: Initial pharmacologic treatment should include metformin and insulin alone or in combination; for metformin, begin with 500 mg daily for 1 week, and titrate by 500 mg once a week over 3–4 week to the maximal dose of 1000 mg twice-daily. Patients who are not metabolically stable require insulin, and if patient fails to reach target HbA1c of < 6.5% within 3–4 months on metformin monotherapy. Bariatric surgery for those who have been unsuccessful with medical therapy alone, but should be done in centers with expertise in this procedure. *Target HbA1c*: < 6.5%
International Diabetes Federation (IDF) & International Society for Pediatric and Adolescent Diabetes (ISPAD)	2011	International	Overweight/obese children > 13 years of age	Not reported	Two abnormal glucose values on OGTT are diagnostic of diabetes on two separate days in asymptomatic individuals	Fasting plasma glucose of ≥126 mg/dL (≥ 7 mmol/L). Casual or 2 h-post challenge plasma glucose of ≥ 200 mg/dL (11.1 mmol/L); or HbA1c > 6.5% (not alone)	*Mainly based on ISPAD guidelines* *Physical activity*: Lifestyle changes in diet and exercise are essential to increase insulin sensitivity *Diet*: Plan should be adapted to cognitive and psychological needs of the child as well as the family’s cultural, ethnic and family traditions *Screen time*: Not reported *Psychosocial issues*: Mental health professionals are needed to support the patient and families. *Medical and surgical therapies*: Metformin – begin with 250 mg daily for 3–4 days, if tolerated increase to 250 mg twice a day, titrate up to a maximal dose of 1000 mg twice a day. Use insulin if needed. Bariatric surgery for adolescents with obesity related co-morbidities, including type 2 diabetes may be considered. This procedure should be performed in a center with an established program collecting outcome data.. *Target HbA1c*: < 7%

*IR* insulin resistance, *AN* Acanthosis nigricans, *BMI* Body mass index, *ICA* Islet cell antibodies, *GAD* anti Glutamic Acid Decarboxylase antibodies, *IA2* Islet Antigen 2 antibodies, *ICA* Islet Cell Antibodies, *IAA* Insulin Autoantibodies, *RPG* Random Plasma Glucose, *FPG* Fasting Plasma Glucose, *PCOS* polycystic ovary syndrome, *ALT* Alanine aminotransferase, *HbA1c* Glycated hemoglobin, *DKA* diabetic ketoacidosis, *OGTT* Oral glucose tolerance test, *NAFLD* Non-alcoholic fatty liver disease, *ADA* American Diabetes Association

*While this paper was under review, a new version of SIGN guidelines was published in November 2017. For the sake of completeness, we used this latest version of the guidelines to report here. In addition, SIGN have published another document specifically reporting the pharmacotherapeutic options for treating T2DM that was referenced in the updated guideline. This latter document was consulted while compiling the recommendations for health care delivery

The guidelines reported on diabetes management in the context of an education program that provides the knowledge and skills needed to manage diabetes, manned by qualified personnel. Though recommendations varied slightly, most guidelines recommended screening every 2 years for T2DM by means of fasting or random glucose tests in the presence of multiple risk factors (i.e., obesity, first-degree relative with T2DM, high-risk ethnic group, signs or symptoms of insulin resistance). Management recommendations included lifestyle changes such as physical activity and limiting screen time, as well as metformin and insulin therapy if needed. Target HbA1c varied but generally was ≤ 7.0% (Table 2).

Most of the CPGs were developed or last updated after 2012. Only one of the included guidelines from the Latvian Diabetes Association was published earlier than 2010, before the development of the AGREE II tool [19]. Of the 21 guidelines, 10 (47.6%) originated from Europe. The majority of the CPGs were developed by diabetes societies or associations (81.0%), whereas the remaining CPGs were developed by government organizations. In terms of guideline scope, 5 of the 21 (23.8%) guidelines were specific to pediatric populations while the remainder of the included guidelines mainly addressed adult T2DM and contained a section for pediatric T2DM.

### Guideline quality scores

The mean scores for individual domains are presented in Table 3. The ICC statistics for the domains varied from 0.85 to 0.95 indicating good to excellent inter-rater reliability for appraisals (Additional file 3). The lowest mean scores were in the “Editorial Independence” domain (mean 44%, SD 33.83) and the “Rigour of Development” domain (mean 46%, SD 29.73), indicating low quality overall for reported guideline development methodology. Scores for “Editorial Independence” were mainly due to lack of reporting and managing conflicts of interest, and less due to involvement of a funding body in guideline development. The domain with the most acceptable score was “Clarity of Presentation” (mean 72%, SD 18.97), as most guidelines distinctly highlighted recommendations for treatment. The mean score for “Scope and Purpose” was 69% (SD 20.22), “Stakeholder Involvement” was 58% (SD 26.41), and “Applicability” was 48% (SD 27.69).

**Table 3:** Quality of included guidelines on six domains using AGREE II tool

Guideline characteristics			AGREE II Domains						
Organization	Year	Country	Scope and Purpose	Stakeholder Involvement	Rigour of Development	Clarity of Presentation	Applicability	Editorial Independence	Recommended for use?
Brazilian Diabetes Society	2015/2016	Brazil	39	28	36	57	34	4	No
Diabetes Canada	2018	Canada	93	94	84	94	70	79	Yes
German Diabetes Association (Deutsche Diabetes Gesellschaft)	Revised 2015	Germany	75	74	67	82	39	92	Yes, with modifications
Hellenic Diabetes Association	2013	Greece	54	28	21	49	24	13	No
Associazione Medici Diabetologi/ Societa Italiana di Diabetologia	2014	Italy	57	54	28	49	29	33	No
Japan Diabetes Society	2013	Japan	58	31	50	56	10	48	No
Latvijas Diabēta Asociācija/Latvijas Endokrinologu Asociācija	2007	Latvia	44	17	2	47	22	0	No
Diabetas Gaires	2014	Lithuania	33	8	2	75	24	0	No
Malaysia Ministry of Health	2015	Malaysia	81	53	45	76	43	75	Yes, with modifications
Netherlands Diabetes Federation	2015	Netherlands	58	51	28	51	17	54	No
Diabetologia Kliniczna	2015	Poland	68	53	18	64	43	6	No
Scottish Intercollegiate Guidelines Network	Updated 2013	Scotland	99	99	96	79	81	65	Yes
Singapore Ministry of Health	2014	Singapore	78	58	30	75	36	6	Yes, with modifications
Endodiab	2016	Slovenia	51	49	41	68	47	19	No
Society for Endocrinology, Metabolism and Diabetes of South Africa	2017	South Africa	60	56	44	74	45	44	Yes, with modifications
Diabetes Association of Thailand	2014	Thailand	50	44	16	51	23	0	No
National Institute for Health and Care Excellence	Updated 2016	United Kingdom	92	74	81	90	68	79	Yes
American Diabetes Association	2016	United States	82	94	86	96	96	88	Yes
American Academy of Pediatrics	2013	United States	99	85	90	100	86	69	Yes
International Society for Pediatric and Adolescent Diabetes	2014	International	89	75	59	97	94	73	Yes
International Diabetes Federation/ International Society for Pediatric and Adolescent Diabetes	2011	International	88	89	67	100	89	79	Yes
Mean (SD)			69 (20.22)	58 (26.41)	46 (29.73)	72 (18.97)	48 (27.69)	44 (33.83)	

AGREE II scoring system: For each domain, scores are rated out on a 7-point scale (1 = strongly disagree, 7 = strongly agree) by individual appraisers. Individual appraiser scores are summed for an overall domain score, which is then scaled to a percentage of the maximum possible score for the domain, with higher scores indicating higher quality. The six domain scores are independent and are not aggregated into a single quality score

The CPG published by the American Academy of Pediatrics and the Scottish Intercollegiate Guidelines Network (SIGN) were the highest ranked for “Scope and Purpose.” The SIGN guideline also ranked highest quality for “Rigour of Development” and “Stakeholder Involvement.” The American Diabetes Association scored highest for “Applicability.” The American Academy of Pediatrics had the highest score for “Clarity of Presentation,” while the German Diabetes Association guidance had the highest score for “Editorial Independence.” Overall, the highest scoring guidelines across domains included the American Academy of Pediatrics, American Diabetes Association, Diabetes Canada (formerly known as the Canadian Diabetes Association), National Institute for Health and Care Excellence (NICE), SIGN, and both international organizations (International Diabetes Federation and International Society for Pediatric and Adolescent Diabetes).

Table 4 shows the comparison of mean scores in each domain for the two most recent versions of CPGs from the American Diabetes Association (2000 and 2016), Brazilian Diabetes Society (2014–2015 and 2015–2016), Diabetes Canada (2013 and 2018), Italian Diabetes Society (2007 and 2014), Singapore Ministry of Health (2006 and 2014), and Society for Endocrinology, Metabolism, and Diabetes of South Africa (2012 and 2017), and the International Society for Pediatric and Adolescent Diabetes (2009 and 2014). The comparison of mean domain scores indicated that more recent versions of guidelines had significantly higher scores than the earlier versions for “Scope and Purpose” (mean 54.43, SD 13.84 for earlier version; mean 71.14, SD 19.66 for latest version; *p* = 0.005) and “Stakeholder Involvement” (mean 43.00, SD 13.98 for earlier version; mean 65.57 SD 23.80 for latest version; *p* = 0.01). There was no significant difference in the mean scores for any other domains between the earlier and most recent versions of the CPGs (*p* values > 0.05). However, despite the lack of statistical significance, there was an increasing trend in mean scores, such that the more recently published versions of guidelines had higher scores for each domain in comparison to the earlier versions.

**Table 4:** Comparison of AGREE II mean domain scores to examine change in overall quality

AGREE II Domain	Earlier version Mean (SD)		Latest version Mean (SD)		*p* value
Scope and Purpose	54.43	13.84	71.14	19.66	0.005*
Stakeholder Involvement	43.00	13.98	65.57	23.80	0.01*
Rigour of Development	39.86	12.29	52.43	24.52	0.20
Clarity of Presentation	66.71	17.40	77.43	19.35	0.11
Applicability	38.14	14.22	57.71	28.73	0.13
Editorial Independence	30.29	34.96	46.71	34.44	0.13

*p < 0.05

Note: Mean domain scores of CPGs from earlier and most recent CPGs available – American Diabetes Association (2000 and 2016), Brazil Diabetes Society (2014–2015 and 2015–2016), Diabetes Canada (2013 and 2018), Italian Diabetes Society (2007 and 2014), Singapore Ministry of Health (2006 and 2014), Society for Endocrinology, Metabolism and Diabetes of South Africa (2012 and 2017) and the International Society for Pediatric and Adolescent Diabetes (2009 and 2014)

## Discussion

### Main findings

Pediatric T2DM is a relatively new disease, and these children will live with this disease for decades longer that adults with T2DM. Therefore, clear evidence-based guidance on the management of diabetes and its comorbidities are paramount to mitigate long-term effects and maintaining good health outcomes. In this review, our goal was to evaluate the current guidelines for management of pediatric T2DM using the AGREE II tool. Findings from this review indicate that the overall quality of CPGs on the management of pediatric T2DM is moderate to low, and can be improved, given that the overall mean scores are below 50% for three of the six domains of the AGREE II tool.

Importantly, when comparing earlier versions of CPGs with the most recent versions, we found significant improvement in the “Scope and Purpose” and “Stakeholder Involvement” mean scores, but no significant change in the mean scores for the remaining domains. Although progress in defining overall purpose and objectives for guidelines and increased involvement of stakeholders indicate improvement over time, there remains a need for improvement in reporting of methodology, appropriate reporting and handling of competing interests, and description of implementation approaches for guideline recommendations. It is important to note, however, that there was an increasing trend in all of the domain scores, such that most recently published versions of the included CPGs had higher scores for each domain in comparison to the earlier versions. Results may not have shown statistical significance given the small sample size (*n* = 7) and especially within the domains that already had somewhat high scores in the earlier versions (i.e., Clarity of Presentation).

The lowest scoring domains were “Rigour of Development,” “Editorial Independence,” and “Applicability.” Low scores in these three domains are especially significant, considering that they directly evaluate the methodological quality, management of conflicts of interest, and details of CPG implementation and monitoring. If guidelines are not effectively developed and reported, then this can impact clinical practice and patient outcomes. Subsequently, if guidelines are developed with no guidance for monitoring or auditing implementation, similar issues can arise.

For “Rigour of Development,” it may be that guidelines are using limited evidence-based approaches in their development methods or are not fully reporting methodological details. The body of evidence for the management for pediatric T2DM is still developing due to its relatively novelty; however, guideline developers should still aim for an evidence-based approach by utilizing available indirect evidence and explicitly stating when clinical expertise was used in the absence of relevant evidence.

However, the concern with a number of CPGs in this area is the lack of reporting and transparency with regard to guideline development methodology. The RIGHT statement is a recently developed resource for the reporting of CPGs [43] that is endorsed by the Enhancing the QUAlity and Transparency Of health Research (EQUATOR) Network, an international initiative to improve quality of reporting in published research. With the application of the RIGHT statement in the CPG development process, improvements in reporting can lead to higher quality of the pediatric T2DM guidelines, especially in areas of “Rigour of Development” and “Editorial Independence.”

For the generally low “Applicability” domain score observed in the included guidelines, it may be that guideline developers intend for the implementation details such as organizational barriers and cost implications to be published separately from the main body of the guideline. However, plans to publish this information should be indicated in the main guideline document. Additionally, conducting implementation analyses with the involvement of health policy experts can aid in the translation of evidence into practice. For instance, involving policy-level work early in the development of the guideline can help create opportunities and collaborations to mobilize public health campaigns for prevention efforts around pediatric T2DM [44]. Early collaboration with public health experts, patients, and parent representatives, followed by involvement of these individuals on guideline panels can also improve “Stakeholder Involvement” and increase the quality of guidelines.

The majority of high-quality guidelines were developed by organizations in countries with potentially more resources and funding for research (e.g., UK, USA, Canada). Additionally, we did not identify any available guidelines for a number of countries. In areas where they may be a lack of funding and resources for guideline development, organizations can also utilize the ADAPTE guideline adaptation process for their institutions and countries, which involves updating and adapting existing high-quality guidelines to local settings rather than to undergo de novo development [45]. This may be useful for pediatric T2DM, since published CPGs from NICE, American Academy of Pediatrics, Diabetes Canada, and others are recommended for use after undergoing quality assessment (Table 3). Also for individual institutions and local organizations considering the development of CPGs for T2DM, it may be favorable to utilize the ADAPTE process to modify a guideline specific to their local context and population.

### Strengths and limitations

This review has several strengths. We conducted a comprehensive search of diabetes and pediatric organizations globally in order to identify eligible CPGs for the review. Four trained assessors evaluated the quality of included guidelines, increasing the reliability of the appraisals. Additionally, we used the AGREE II tool, which has established reliability and validity, to conduct quality assessment [13, 46].

A limitation of this review was the use of Google Translate to assess guidelines that were not available in English. We utilized this method because it was unfeasible due to time and resources to identify four independent bilingual appraisers that were fluent for the included language(s) as well as English, could undergo AGREE II training, and conduct appraisal for the guidelines. Therefore, we used the back-translate approach, from the language of publication to English and back to the language of publication, to determine the overall similarity of the translation to the original document. When using the back-translation approach, we did not note any substantial differences in the text—however, this may be due to use of the same software (Google Translate) for original and back-translation. We note that Google Translate may not be the ideal method for all of our included languages, specifically for the Thai guideline; however, we aimed to maintain a broad scope for the review and determined that it was the best and most feasible method [11, 47]. Also in order to conduct a broad review, we included CPGs with broader scope that incorporated a section dedicated to pediatric T2DM. This may have limited the review if guidelines with different primary objectives (i.e., type 1 diabetes guidelines, guidelines for adult diabetes) received a lower AGREE II score when evaluated for the quality of recommendations pertaining to pediatric patients. However, we appraised all included CPGs for overall methodology and development irrespective of the pediatric recommendations.

### Clinical and research implications

This review has major implications as it directly affects clinical practice. The rise in T2DM in children and youth is a serious public health concern, and more patients will be transitioned to adult care having been diagnosed with T2DM as children. These patients will deal with the burden of living with this chronic disease and its comorbidities longer than those diagnosed with T2DM during adulthood. It is important that rigourous methods are used to develop CPGs and reported with transparency for the knowledge end users. Given that pediatric T2DM has emerged only recently, it is especially imperative that clinicians have adequate guidance for its management.

The quality of CPGs may improve with the widespread implementation of reporting guidelines. This trend in improvement has been observed within other types of studies, such as randomized controlled trials [48]. Along with quality of reporting, direct improvements can be made to these guidelines by (1) involving relevant stakeholders, including patient representatives and experts throughout the guideline development process, (2) reporting the guideline development methodology in detail, and (3) discussing barriers and facilitators for implementation of the guideline recommendations. Given that management recommendations for pediatric T2DM involve behavioral changes and self-driven interventions as well as pharmacotherapy, it is especially important to include patients’ and parents’ perspectives to improve guideline development and implementation.

## Conclusions

Overall, two thirds of the pediatric T2DM guidelines were rated moderate to low quality and the remaining third ranked higher in quality. There are areas requiring significant improvement such as rigour of development, editorial independence, applicability including tools and barriers for implementation, and stakeholder involvement. Deficiencies noted in guideline development methodology and quality of reporting can be improved by following published reporting statements. Also comparisons of the AGREE II scores between different CPGs suggest that some organizations and societies may have greater access to the substantial resources and time needed for de novo development of CPGs. It may be useful for other organizations to use a guideline adaptation process to achieve high-quality guidance for clinicians in a given setting.

## Additional files

Additional file 1: PRISMA 2009 Checklist. (DOC 64 kb)

Additional file 2: Pediatric Type 2 Diabetes Clinical Practice Guidelines Search strategy. (DOCX 74 kb)

Additional file 3: ICC Statistics. (DOCX 12 kb)
