# Supplementary material for: The quality of clinical practice guidelines for management of pediatric type 2 diabetes mellitus: a systematic review using the AGREE II instrument
Source: Syst Rev. 2018 Nov 15;7:193. doi: 10.1186/s13643-018-0843-1 (PMC6238336; doi:10.1186/s13643-018-0843-1)
Supplement: Supplementary file 2 — Pediatric Type 2 Diabetes Clinical Practice Guidelines Search strategy. (DOCX 74 kb) [file 13643_2018_843_MOESM2_ESM.docx]

**Supplementary Material –**

Pediatric Type 2 Diabetes Clinical Practice Guidelines Search strategy

Note: For TRIP and National Guideline Clearinghouse, broad searches were used.

**National Guideline Clearinghouse** – screened all citations retrieved upon searching ‘type 2 diabetes’, limited to children 2-12 yrs, adolescents 13-18 yrs

**TRIP** – screened all citations retrieved upon searching “type 2 diabetes and (children and adolescents)”

**MEDLINE**

1 practice guideline/

2 guideline/

3 guideline.pt.

4 guideline*.mp.

5 exp consensus development conference/

6 consensus development conference*.mp.

7 consensus statement*.mp.

8 or/1-7

9 Diabetes Mellitus/

10 exp Diabetes Mellitus, Type 2/

11 exp Diabetes Mellitus, Type 1/

12 diabet*.mp.

13 IDDM.mp.

14 NIDDM.mp.

15 (T1DM or T2DM or T1D or T2D).mp.

16 ((noninsulin or non insulin or insulin) adj2 depend*).mp.

17 or/9-16

18 Adolescent/

19 child/

20 child, preschool/

21 child*.mp.

22 adolescen*.mp.

23 teen*.mp.

24 youth*.mp.

25 p?ediatric*.mp.

26 pediatrics/

27 or/18-26

28 8 and 17 and 27

29 remove duplicates from 28

**EMBASE**

1 practice guideline/

2 consensus development/

3 guideline*.mp.

4 consensus statement*.mp.

5 consensus development conference*.mp.

6 or/1-5

7 diabetes mellitus/

8 insulin dependent diabetes mellitus/

9 non insulin dependent diabetes mellitus/

10 diabet*.mp.

11 IDDM.mp.

12 NIDDM.mp.

13 (T1DM or T2DM or T1D or T2D).mp.

14 ((noninsulin or non insulin or insulin) adj2 depend*).mp.

15 or/7-14

16 adolescent/

17 child/

18 preschool child/

19 adolescen*.mp.

20 teen*.mp.

21 youth*.mp.

22 child*.mp.

23 pediatrics/

24 p?ediatric*.mp.

25 or/16-24

26 6 and 15 and 25

27 remove duplicates from 26

**CINAHL**

S26 S6 AND S15 AND S25

S25 S16 OR S17 OR S18 OR S19 OR S20 OR
S21 OR S22 OR S23
OR S24

S24 pediatric* OR
paediatric*

S23 (MH "Pediatrics")

S22 youth*

S21 teen*

S20 adolescen*

S19 child*

S18 (MH "Child, Preschool")

S17 (MH "Child")

S16 (MH "Adolescence")

S15 S7 OR S8 OR S9 OR S10 OR S11 OR S12
OR S13 OR S14

S14 (noninsulin or noninsulin or insulin) N2
depend*

S13 T1DM or T2DM or T1D
or T2D

S12 NIDDM

S11 IDDM

S10 diabet*

S9 (MH "Diabetes Mellitus, Type 2")

S8 (MH "Diabetes Mellitus,
Type 1+")

S7 (MH "Diabetes
Mellitus")

S6 S1 OR S2 OR S3 OR S4 OR S5

S5 "consensus statement*"

S4 "consensus
development
conference*"

S3 PT practice guidelines

S2 guideline*

S1 (MH "Practice
Guidelines")
