# Supplementary material for: The quality of clinical practice guidelines for management of pediatric type 2 diabetes mellitus: a systematic review using the AGREE II instrument
Source: Syst Rev. 2018 Nov 15;7:193. doi: 10.1186/s13643-018-0843-1 (PMC6238336; doi:10.1186/s13643-018-0843-1)
Supplement: Supplementary file 3 — ICC Statistics. (DOCX 12 kb) [file 13643_2018_843_MOESM3_ESM.docx]

| **Domain** | **ICC Statistic (95% CI)** |
| --- | --- |
| Domain 1 | 0.90 (0.80 – 0.95) |
| Domain 2 | 0.91 (0.82 – 0.96) |
| Domain 3 | 0.97 (0.94 – 0.99) |
| Domain 4 | 0.86 (0.72 – 0.93) |
| Domain 5 | 0.85 (0.72 – 0.93) |
| Domain 6 | 0.95 (0.90 – 0.98) |

Additional File 3 – ICC Statistics

ICC Statistic calculated using one-way random effects model. Result of average measures reported. Measuring reliability across 4 raters for the 21 guidelines.
